# Supplementary material for: MRI-based anatomical characterisation of lower-limb muscles in older women
Source: PLoS One. 2020 Dec 1;15(12):e0242973. doi: 10.1371/journal.pone.0242973 (PMC7707470; doi:10.1371/journal.pone.0242973)
Supplement: S1 Table — (DOCX) [file pone.0242973.s001.docx]

|  | Subject code | Height [cm] | Weight [kg] | BMI | Age [year] |
| --- | --- | --- | --- | --- | --- |
| Subject 1 | **MC17** | 164.0 | 61.4 | 22.8 | 70.5 |
| Subject 2 | **MC18** | 156.0 | 75.8 | 31.1 | 64.1 |
| Subject 3 | **MC19** | 160.5 | 78.6 | 30.5 | 73.0 |
| Subject 4 | **MC20** | 158.5 | 69.4 | 27.6 | 67.4 |
| Subject 5 | **MC22** | 160.0 | 66.3 | 25.9 | 72.2 |
| Subject 6 | **MC24** | 160.5 | 58.8 | 22.8 | 65.3 |
| Subject 7 | **MC25** | 163.5 | 56.8 | 21.2 | 74.6 |
| Subject 8 | **MC26** | 158.5 | 64.2 | 25.6 | 68.5 |
| Subject 9 | **MC27** | 155.0 | 67.0 | 27.9 | 61.8 |
| Subject 10 | **MC28** | 154.0 | 60.0 | 25.3 | 83.0 |
| Subject 11 | **MC29** | 160.4 | 78.0 | 30.3 | 59.1 |
| Mean |  | 159.2 | 66.9 | 26.5 | 69.0 |
| SD |  | 3.2 | 7.7 | 3.4 | 6.7 |

Table 1 – Anthropometric data
