# Supplementary material for: MRI-based anatomical characterisation of lower-limb muscles in older women
Source: PLoS One. 2020 Dec 1;15(12):e0242973. doi: 10.1371/journal.pone.0242973 (PMC7707470; doi:10.1371/journal.pone.0242973)
Supplement: S2 Table — Maximum coefficient of variation (CoV) across the three datasets is reported. (DOCX) [file pone.0242973.s002.docx]

**Inter-operator repeatability of muscle volumes [cm^3^]**

| Muscles | Subject 1 | | | Subject 2 | | | Subject 3 | | | Max CoV |
| --- | --- | --- | --- | --- | --- | --- | --- | --- | --- | --- |
|  | **OP1** | **OP2** | **OP3** | **OP1** | **OP2** | **OP3** | **OP1** | **OP2** | **OP3** |  |
| Adductor brevis | 65.5 | 48.3 | 55.4 | 48.2 | 59.6 | 56 | 61.4 | 79.1 | 50.3 | 22.8 |
| Adductor longus | 75.9 | 95.6 | 108.7 | 66.8 | 78.7 | 71.7 | 81.1 | 91.6 | 77.6 | 17.7 |
| Adductor magnus | 291.2 | 316.1 | 294.7 | 313.3 | 329.4 | 352.3 | 322.2 | 352.2 | 346.8 | 5.9 |
| Biceps femoris short head | 51.5 | 42.2 | 46.9 | 87.2 | 82.7 | 84.9 | 61.9 | 60.5 | 61.2 | 9.9 |
| Biceps femoris long head | 131.7 | 143.7 | 143.2 | 119.6 | 135 | 124.8 | 110.7 | 120.8 | 104 | 7.6 |
| Gastrocnemius lateralis | 96.5 | 99.5 | 95.8 | 59.9 | 60.3 | 65 | 97.8 | 102.8 | 99.4 | 4.6 |
| Gastrocnemius medialis | 179 | 187.7 | 184.5 | 139.2 | 146.5 | 149.2 | 147.5 | 158 | 160.6 | 4.5 |
| Gluteus maximus | 561.4 | 617.1 | 592.2 | 409.1 | 447.4 | 469.9 | 574.1 | 559.7 | 577.3 | 7 |
| Gluteus medius | 236.8 | 257.8 | 271.7 | 208.7 | 220.1 | 255.1 | 256.7 | 293.9 | 262.7 | 10.6 |
| Gluteus minimus | 62.1 | 75.3 | 67.1 | 56.9 | 64.5 | 75.9 | 33.6 | 42.9 | 37.3 | 14.5 |
| Gracilis | 48.1 | 52.7 | 57.3 | 40.6 | 40.4 | 39.3 | 43.3 | 52.5 | 38.2 | 16.2 |
| Iliacus | 109 | 119.5 | 123.7 | 123.4 | 119.2 | 138.4 | 117.1 | 112.1 | 125.6 | 7.9 |
| Peroneus brevis | 18.6 | 20.8 | 43.3 | 32.7 | 29.9 | 31.8 | 33.6 | 42.8 | 55.4 | 49.6 |
| Peroneus longus | 37.6 | 42.9 | 14.3 | 25.4 | 24 | 21.5 | 39.4 | 36.4 | 23.2 | 48.1 |
| Psoas | 97.1 | 108.6 | 99.4 | 70.1 | 78.7 | 76.1 | 51.6 | 57.6 | 49.7 | 7.8 |
| Rectus femoris | 103.7 | 101.1 | 110.1 | 123.6 | 135.7 | 138.8 | 132.3 | 152.2 | 144.4 | 7 |
| Sartorius | 77.7 | 90.2 | 95.1 | 69 | 77.2 | 71.7 | 96.9 | 98.5 | 88.9 | 10.2 |
| Semimembranosus | 165.2 | 163.8 | 167 | 157 | 167.4 | 163.1 | 88.2 | 108.5 | 94.5 | 10.7 |
| Semitendinosus | 81.8 | 92.5 | 87.9 | 118.5 | 130.6 | 120 | 109.4 | 119.5 | 104.5 | 6.9 |
| Soleus | 346.3 | 373 | 369 | 297.3 | 345.1 | 347.9 | 338.1 | 341 | 347.1 | 8.6 |
| Tensor fasciae latae | 33.9 | 43.2 | 41.4 | 28.9 | 32.5 | 33.3 | 41.3 | 48.5 | 45.1 | 12.5 |
| Tibialis anterior | 110.6 | 100.6 | 116.6 | 82.2 | 120.9 | 138.5 | 83.1 | 119.1 | 88.6 | 25.3 |
| Tibialis posterior | 76.2 | 70.9 | 81.4 | 60.9 | 72.4 | 77.4 | 65.8 | 81.1 | 68.4 | 12 |
| Vastus intermedius | 251.6 | 266.3 | 272.4 | 233.9 | 250.2 | 233.9 | 199.6 | 223.5 | 225.1 | 6.6 |
| Vastus lateralis | 321.1 | 326 | 326.4 | 286.4 | 348.2 | 328.3 | 303 | 335.4 | 339.2 | 9.8 |
| Vastus medialis | 182 | 195.9 | 196.2 | 208.1 | 206.8 | 206.5 | 205.6 | 207 | 201.2 | 4.2 |

Table 2 – Right-limb muscle volumes segmented by three operators for three randomly selected subjects. Maximum coefficient of variation (CoV) across the three datasets is reported.
