# Supplementary material for: MRI-based anatomical characterisation of lower-limb muscles in older women
Source: PLoS One. 2020 Dec 1;15(12):e0242973. doi: 10.1371/journal.pone.0242973 (PMC7707470; doi:10.1371/journal.pone.0242973)
Supplement: S3 Table — Coefficient of variation (CoV) across the three repetitions is reported. (DOCX) [file pone.0242973.s003.docx]

**Intra-operator repeatability of muscle volumes [cm^3^]**

| Muscles | Subject 1 | | | CoV |
| --- | --- | --- | --- | --- |
|  | **REP1** | **REP2** | **REP3** |  |
| Adductor brevis | 54.7 | 47.7 | 54.2 | 7.5 |
| Adductor longus | 73.1 | 81.7 | 74.6 | 6.0 |
| Adductor magnus | 362.3 | 342.0 | 339.8 | 3.6 |
| Biceps femoris caput breve | 54.8 | 57.4 | 60.2 | 4.7 |
| Gastrocnemius lateralis | 71.4 | 79.8 | 80.0 | 6.4 |
| Gastrocnemius medialis | 114.1 | 126.1 | 129.6 | 6.6 |
| Gluteus maximus | 626.2 | 616.8 | 641.9 | 2.0 |
| Gluteus medius | 238.5 | 260.5 | 262.9 | 5.3 |
| Gluteus minimus | 37.7 | 58.6 | 52.0 | 21.6 |
| Gracilis | 45.0 | 45.6 | 43.3 | 2.7 |
| Iliacus | 103.5 | 109.0 | 105.8 | 2.6 |
| Peroneus brevis | 28.8 | 33.3 | 32.4 | 7.6 |
| Peroneus longus | 34.7 | 32.5 | 40.1 | 10.9 |
| Rectus femoris | 106.4 | 114.8 | 118.9 | 5.6 |
| Sartorius | 91.8 | 89.1 | 92.5 | 2.0 |
| Semimembranosus | 80.9 | 92.3 | 90.2 | 6.9 |
| Semitendinosus | 115.8 | 108.1 | 104.6 | 5.2 |
| Soleus | 279.0 | 277.4 | 250.6 | 5.9 |
| Tensor fasciae latae | 49.4 | 49.9 | 50.5 | 1.1 |
| Tibialis anterior | 78.7 | 77.7 | 84.0 | 4.2 |
| Tibialis posterior | 66.8 | 72.9 | 79.8 | 8.9 |
| Vastus intermedius | 165.9 | 162.4 | 163.3 | 1.1 |
| Vastus lateralis | 312.2 | 310.3 | 317.8 | 1.2 |

Table 3 - Right-limb muscle volumes segmented three times by one operator. Coefficient of variation (CoV) across the three repetitions is reported.
