# Supplementary material for: MRI-based anatomical characterisation of lower-limb muscles in older women
Source: PLoS One. 2020 Dec 1;15(12):e0242973. doi: 10.1371/journal.pone.0242973 (PMC7707470; doi:10.1371/journal.pone.0242973)
Supplement: S5 Table — (DOCX) [file pone.0242973.s005.docx]

**Muscle length [mm]**

| Muscle | Subject 1 | | Subject 2 | | Subject 3 | | Subject 4 | | Subject 5 | | Subject 6 | | Subject 7 | | Subject 8 | | Subject 9 | | Subject 10 | | Subject 11 | |  |  |  |
| --- | --- | --- | --- | --- | --- | --- | --- | --- | --- | --- | --- | --- | --- | --- | --- | --- | --- | --- | --- | --- | --- | --- | --- | --- | --- |
|  | **Right** | **Left** | **Right** | **Left** | **Right** | **Left** | **Right** | **Left** | **Right** | **Left** | **Right** | **Left** | **Right** | **Left** | **Right** | **Left** | **Right** | **Left** | **Right** | **Left** | **Right** | **Left** | **Mean** | **SD** | **CoV** |
| Adductor brevis | 202 | 218 | 183 | 171 | 228 | 177 | 235 | 245 | 182 | 183 | 196 | 203 | 200 | 204 | 215 | 226 | 192 | 178 | 220 | 258 | 221 | 243 | 208 | 25 | 12 |
| Adductor longus | 234 | 237 | 246 | 234 | 263 | 267 | 295 | 291 | 272 | 297 | 280 | 266 | 237 | 260 | 283 | 268 | 236 | 212 | 286 | 263 | 232 | 269 | 260 | 24 | 9 |
| Adductor magnus | 363 | 356 | 372 | 377 | 373 | 390 | 365 | 374 | 342 | 357 | 361 | 313 | 364 | 359 | 322 | 307 | 297 | 279 | 304 | 296 | 389 | 396 | 348 | 35 | 10 |
| Biceps femoris short head | 208 | 202 | 227 | 224 | 253 | 258 | 240 | 258 | 230 | 242 | 281 | 281 | 242 | 254 | 207 | 217 | 227 | 223 | 234 | 221 | 210 | 220 | 234 | 22 | 10 |
| Biceps femoris long head | 291 | 298 | 280 | 275 | 302 | 324 | 257 | 261 | 250 | 254 | 273 | 259 | 293 | 283 | 282 | 261 | 292 | 283 | 227 | 262 | 298 | 296 | 277 | 22 | 8 |
| Gluteus maximus | 255 | 259 | 206 | 211 | 286 | 247 | 202 | 202 | 191 | 211 | 214 | 208 | 194 | 185 | 239 | 266 | 253 | 266 | 225 | 261 | 265 | 263 | 232 | 31 | 13 |
| Gluteus medius | 279 | 273 | 268 | 266 | 321 | 325 | 255 | 262 | 263 | 280 | 264 | 265 | 269 | 256 | 289 | 269 | 307 | 292 | 250 | 298 | 330 | 327 | 282 | 25 | 9 |
| Gracilis | 280 | 255 | 278 | 267 | 271 | 275 | 281 | 264 | 278 | 254 | 279 | 275 | 286 | 283 | 280 | 256 | 285 | 274 | 294 | 269 | 290 | 272 | 275 | 11 | 4 |
| Iliacus | 341 | 343 | 340 | 356 | 360 | 358 | 388 | 399 | 365 | 372 | 379 | 380 | 343 | 312 | 377 | 370 | 332 | 337 | 322 | 357 | 342 | 333 | 355 | 22 | 6 |
| Rectus femoris | 230 | 242 | 231 | 228 | 246 | 256 | 296 | 346 | 260 | 236 | 294 | 300 | 275 | 258 | 253 | 233 | 226 | 221 | 264 | 225 | 223 | 238 | 254 | 32 | 12 |
| Sartorius | 351 | 323 | 314 | 318 | 319 | 304 | 335 | 340 | 354 | 331 | 318 | 318 | 320 | 326 | 349 | 331 | 308 | 307 | 314 | 306 | 325 | 318 | 324 | 14 | 4 |
| Semimembranosus | 595 | 586 | 576 | 581 | 591 | 592 | 617 | 626 | 609 | 595 | 590 | 593 | 610 | 593 | 562 | 566 | 589 | 594 | 585 | 590 | 572 | 585 | 591 | 15 | 3 |
| Semitendinosus | 302 | 317 | 269 | 273 | 318 | 309 | 287 | 278 | 217 | 232 | 328 | 323 | 297 | 285 | 251 | 283 | 294 | 301 | 257 | 276 | 282 | 289 | 285 | 28 | 10 |
| Tensor fasciae latae | 321 | 330 | 305 | 332 | 322 | 330 | 342 | 322 | 310 | 325 | 367 | 371 | 362 | 351 | 342 | 365 | 292 | 285 | 292 | 289 | 316 | 283 | 325 | 27 | 8 |
| Vastus intermedius | 374 | 373 | 395 | 374 | 392 | 389 | 353 | 368 | 316 | 318 | 382 | 380 | 375 | 354 | 362 | 336 | 366 | 357 | 352 | 379 | 365 | 349 | 364 | 21 | 6 |
| Vastus lateralis | 183 | 170 | 182 | 196 | 217 | 217 | 173 | 166 | 203 | 202 | 206 | 183 | 163 | 170 | 208 | 212 | 177 | 183 | 182 | 194 | 197 | 188 | 190 | 16 | 9 |
| Vastus medialis | 311 | 320 | 338 | 273 | 298 | 317 | 322 | 330 | 374 | 350 | 330 | 318 | 301 | 299 | 341 | 352 | 364 | 327 | 284 | 286 | 314 | 351 | 323 | 26 | 8 |
| Gastrocnemius lateralis | 202 | 218 | 183 | 171 | 228 | 177 | 235 | 245 | 182 | 183 | 196 | 203 | 200 | 204 | 215 | 226 | 192 | 178 | 220 | 258 | 221 | 243 | 208 | 25 | 12 |
| Gastrocnemius medialis | 234 | 237 | 246 | 234 | 263 | 267 | 295 | 291 | 272 | 297 | 280 | 266 | 237 | 260 | 283 | 268 | 236 | 212 | 286 | 263 | 232 | 269 | 260 | 24 | 9 |
| Peroneus brevis | 363 | 356 | 372 | 377 | 373 | 390 | 365 | 374 | 342 | 357 | 361 | 313 | 364 | 359 | 322 | 307 | 297 | 279 | 304 | 296 | 389 | 396 | 348 | 35 | 10 |
| Soleus | 291 | 298 | 280 | 275 | 302 | 324 | 257 | 261 | 250 | 254 | 273 | 259 | 293 | 283 | 282 | 261 | 292 | 283 | 227 | 262 | 298 | 296 | 277 | 22 | 8 |
| Tibialis anterior | 255 | 259 | 206 | 211 | 286 | 247 | 202 | 202 | 191 | 211 | 214 | 208 | 194 | 185 | 239 | 266 | 253 | 266 | 225 | 261 | 265 | 263 | 232 | 31 | 13 |
| Tibialis posterior | 279 | 273 | 268 | 266 | 321 | 325 | 255 | 262 | 263 | 280 | 264 | 265 | 269 | 256 | 289 | 269 | 307 | 292 | 250 | 298 | 330 | 327 | 282 | 25 | 9 |

Table 5 – Right and left length of the muscles segmented in the lower limbs of the eleven subjects enrolled in the study Mean, standard deviation (SD) and coefficient of variation (CoV) are reported .
