# Supplementary material for: MRI-based anatomical characterisation of lower-limb muscles in older women
Source: PLoS One. 2020 Dec 1;15(12):e0242973. doi: 10.1371/journal.pone.0242973 (PMC7707470; doi:10.1371/journal.pone.0242973)
Supplement: S6 Table — Mean and mean and SD PCSA are reported for Ward et al. 2009 and Handsfield et al. 2014, respectively. (DOCX) [file pone.0242973.s006.docx]

**Muscle PCSA [cm^2^]**

|  | This study | | | | | | | | | | | | | | | | | | | | | | Charles et al | | | Ward  et al | Handsfield  et al | |
| --- | --- | --- | --- | --- | --- | --- | --- | --- | --- | --- | --- | --- | --- | --- | --- | --- | --- | --- | --- | --- | --- | --- | --- | --- | --- | --- | --- | --- |
|  | **Sbj1** | | **Sbj2** | | **Sbj3** | | **Sbj4** | | **Sbj5** | | **Sbj6** | | **Sbj7** | | **Sbj8** | | **Sbj9** | | **Sbj10** | | **Sbj11** | |  |  |  |  |  |  |
|  | **R** | **L** | **R** | **L** | **R** | **L** | **R** | **L** | **R** | **L** | **R** | **L** | **R** | **L** | **R** | **L** | **R** | **L** | **R** | **L** | **R** | **L** | **Smp 1** | **Smp 2** | **Smp 3** | **Mean** | **Mean** | **SD** |
| Adductor brevis | 4.5 | 3.9 | 5.0 | 4.3 | 5.0 | 6.2 | 2.9 | 2.9 | 4.7 | 4.2 | 5.2 | 5.2 | 4.3 | 3.5 | 2.6 | 2.7 | 4.4 | 5.4 | 3.6 | 3.5 | 4.7 | 4.0 | 5.1 | 3.4 | 4.9 | 5.0 | 9.7 | 1.9 |
| Adductor longus | 5.9 | 5.3 | 6.8 | 7.2 | 4.5 | 6.0 | 4.9 | 4.8 | 5.1 | 5.1 | 8.4 | 7.6 | 5.6 | 5.1 | 4.9 | 5.4 | 6.7 | 7.2 | 6.4 | 6.2 | 7.7 | 6.6 | 6.7 | 6.2 | 6.5 | 6.6 | 15.4 | 3.6 |
| Adductor magnus | 31.6 | 32.4 | 27.3 | 22.9 | 26.1 | 22.9 | 22.3 | 21.8 | 24.3 | 23.0 | 26.3 | 24.3 | 22.8 | 22.4 | 23.4 | 19.1 | 29.0 | 26.8 | 23.8 | 27.9 | 32.9 | 26.1 | 26.8 | 50.1 | 31.0 | 21.3 | 45.5 | 8.5 |
| Biceps femoris long head | 15.7 | 13.9 | 15.7 | 17.3 | 14.4 | 14.8 | 12.9 | 11.6 | 11.2 | 11.6 | 12.6 | 14.3 | 14.5 | 15.1 | 11.4 | 11.8 | 16.6 | 16.6 | 15.0 | 15.1 | 16.8 | 16.4 | 13.1 | 12.3 | 16.3 | 11.5 | 25.9 | 4.9 |
| Biceps femoris short head | 5.1 | 4.3 | 3.7 | 3.4 | 5.9 | 5.5 | 2.7 | 3.1 | 4.5 | 5.8 | 4.4 | 3.8 | 6.8 | 7.0 | 3.4 | 3.7 | 5.1 | 4.7 | 6.0 | 5.7 | 7.5 | 5.4 | 4.1 | 10.7 | 11.1 | 5.2 | 7.8 | 1.8 |
| Gastrocnemius lateralis | 12.6 | 11.5 | 15.3 | 11.6 | 10.4 | 8.4 | 12.4 | 11.3 | 9.3 | 10.7 | 11.0 | 12.0 | 12.1 | 12.0 | 13.4 | 11.6 | 10.1 | 13.5 | 13.4 | 13.9 | 13.5 | 14.1 | 13.1 | 11.8 | 12.0 | 36.0 | 46.8 | 8.7 |
| Gastrocnemius medialis | 26.6 | 28.3 | 36.3 | 35.2 | 28.8 | 30.6 | 27.2 | 32.0 | 26.3 | 27.3 | 26.8 | 28.6 | 30.7 | 28.6 | 26.1 | 27.3 | 32.3 | 32.2 | 25.8 | 26.1 | 35.9 | 36.6 | - | - | - | 36.1 | 45.6 | 10.2 |
| Gluteus maximus | 26.2 | 27.3 | 34.8 | 33.6 | 30.8 | 28.5 | 24.7 | 24.8 | 31.8 | 27.3 | 21.4 | 22.1 | 21.6 | 20.9 | 23.3 | 21.3 | 27.4 | 25.6 | 27.8 | 27.3 | 35.5 | 34.7 | - | - | - | 2.2 | 4.7 | 1 |
| Gluteus medius | 24.2 | 21.0 | 25.0 | 26.0 | 28.4 | 29.2 | 18.8 | 20.5 | 23.1 | 20.0 | 22.6 | 26.3 | 18.6 | 19.9 | 17.7 | 17.5 | 23.8 | 23.3 | 26.2 | 25.8 | 39.1 | 34.4 | 17.8 | 17.9 | 20.3 | 10.2 | 12.4 | 2.5 |
| Gracilis | 1.3 | 1.4 | 2.8 | 2.1 | 1.5 | 1.9 | 0.6 | 1.0 | 1.5 | 1.0 | 1.8 | 1.8 | 1.8 | 1.6 | 1.1 | 0.9 | 1.8 | 1.8 | 1.5 | 1.5 | 2.5 | 2.6 | 5.3 | 5.8 | 3.6 | 9.9 | 23 | 6 |
| Iliacus | 4.5 | 5.4 | 6.8 | 6.9 | 5.7 | 5.6 | 5.5 | 5.5 | 5.4 | 5.4 | 6.1 | 5.8 | 6.5 | 6.5 | 5.8 | 5.5 | 6.6 | 6.1 | 6.4 | 6.2 | 8.6 | 7.1 | - | - | - | 21.4 | 50.1 | 10 |
| Peroneus brevis | 9.2 | 6.5 | 9.4 | 8.2 | 8.9 | 7.5 | 6.0 | 8.7 | 5.9 | 6.1 | 5.9 | 7.3 | 7.4 | 6.7 | 7.0 | 5.4 | 5.7 | 4.4 | 8.1 | 7.8 | 9.1 | 7.6 | - | - | - | 5.0 | - | - |
| Rectus femoris | 15.6 | 17.1 | 10.4 | 19.2 | 16.7 | 18.1 | 13.3 | 14.4 | 15.3 | 14.6 | 22.6 | 23.6 | 19.0 | 18.0 | 19.3 | 16.9 | 17.5 | 16.1 | 19.0 | 20.6 | 23.0 | 19.6 | 22.7 | 22.0 | 26.1 | 13.9 | 34.8 | 7.4 |
| Sartorius | 1.1 | 1.0 | 1.8 | 1.5 | 2.0 | 1.4 | 1.2 | 1.0 | 1.5 | 1.5 | 1.7 | 1.5 | 1.1 | 1.3 | 1.0 | 1.0 | 1.6 | 1.5 | 1.9 | 1.8 | 3.2 | 2.9 | 3.2 | 3.0 | 3.2 | 1.9 | 3.4 | 0.8 |
| Semimembranosus | 16.8 | 16.4 | 23.4 | 22.7 | 16.6 | 20.1 | 15.0 | 17.4 | 18.8 | 15.5 | 13.7 | 14.9 | 21.6 | 23.0 | 17.8 | 15.3 | 21.9 | 22.9 | 16.0 | 13.3 | 18.5 | 17.7 | 8.5 | 28.6 | 21.2 | 19.1 | 37.8 | 9.1 |
| Semitendinosus | 3.4 | 4.1 | 7.2 | 7.2 | 4.4 | 3.8 | 4.3 | 4.9 | 5.1 | 4.0 | 5.9 | 5.2 | 4.3 | 5.2 | 2.8 | 2.4 | 4.7 | 4.4 | 4.7 | 5.8 | 6.7 | 7.0 | 12.8 | 15.7 | 15.4 | 4.9 | 9.3 | 2.3 |
| Soleus | 74.4 | 69.7 | 92.3 | 89.6 | 94.1 | 88.0 | 83.2 | 90.8 | 74.1 | 79.2 | 68.0 | 69.6 | 73.9 | 76.3 | 67.0 | 71.5 | 89.4 | 88.3 | 84.7 | 81.2 | 125.1 | 134.5 | 80.3 | 45.8 | 85.2 | 58.8 | 124.1 | 24.9 |
| Tensor fasciae latae | 2.5 | 2.0 | 2.9 | 2.7 | 2.3 | 2.4 | 1.0 | 1.3 | 1.7 | 1.6 | 1.6 | 1.8 | 2.2 | 1.7 | 2.8 | 3.2 | 2.0 | 1.9 | 3.2 | 2.1 | 2.3 | 2.8 | - | - | - | 2.5 | 4 | 1.3 |
| Tibialis anterior | 9.9 | 11.5 | 11.3 | 13.1 | 11.7 | 11.9 | 9.1 | 9.9 | 8.8 | 9.0 | 8.8 | 8.8 | 9.2 | 10.2 | 12.0 | 10.5 | 11.2 | 12.5 | 11.6 | 10.8 | 16.0 | 12.0 | 19.8 | 11.3 | 17.0 | 11.1 | 15.8 | 2.9 |
| Tibialis posterior | 18.5 | 18.5 | 22.2 | 20.6 | 21.7 | 21.8 | 23.1 | 20.9 | 14.7 | 15.8 | 18.7 | 19.1 | 15.2 | 18.3 | 22.9 | 24.1 | 20.7 | 22.3 | 21.0 | 17.8 | 29.0 | 32.9 | - | - | - | 14.8 | 28.4 | 4.7 |
| Vastus intermedius | 22.6 | 26.0 | 36.7 | 38.2 | 27.6 | 34.6 | 30.7 | 27.6 | 25.7 | 25.9 | 28.0 | 24.8 | 27.9 | 23.2 | 26.7 | 26.7 | 28.6 | 27.7 | 23.8 | 21.1 | 38.2 | 29.2 | 14.2 | 31.7 | 38.8 | 16.8 | 39 | 8 |
| Vastus lateralis | 18.8 | 20.0 | 24.8 | 23.7 | 25.1 | 23.7 | 17.6 | 21.2 | 14.0 | 18.1 | 19.5 | 21.1 | 21.9 | 19.4 | 16.4 | 16.3 | 23.4 | 22.5 | 20.3 | 19.8 | 30.4 | 26.5 | 18.6 | 28.2 | 61.9 | 37.0 | 59.3 | 12.1 |
| Vastus medialis | 22.6 | 21.3 | 29.9 | 26.7 | 32.3 | 31.6 | 27.2 | 30.7 | 28.9 | 28.3 | 28.2 | 26.0 | 25.5 | 24.3 | 26.2 | 22.9 | 24.5 | 24.1 | 24.2 | 24.9 | 33.5 | 27.0 | 23.6 | 35.0 | 25.9 | 23.7 | 59.1 | 11.4 |

Table 6 –Physiological cross-sectional areas (PCSAs) measured for the eleven subjects enrolled in our study (for right and left muscles) and three cadavers included in Charles et al., 2019. Mean and mean and SD PCSA are reported for Ward et al. 2009 and Handsfield et al. 2014, respectively.

**References**

1. Charles JP, Moon C-H, Anderst WJ. Determining subject-specific lower-limb muscle architecture data for musculoskeletal models using diffusion tensor imaging. Journal of Biomechanical Engineering. 2019;141(6).
2. Handsfield GG, Meyer CH, Hart JM, Abel MF, Blemker SS. Relationships of 35 lower limb muscles to height and body mass quantified using MRI. Journal of Biomechanical Engineering. 2014;47(3):631-8.

Ward SR, Lieber RL. Density and hydration of fresh and fixed human skeletal muscle. Journal of Biomechanics. 2005;38(11):2317-20.
