# Supplementary material for: MRI-based anatomical characterisation of lower-limb muscles in older women
Source: PLoS One. 2020 Dec 1;15(12):e0242973. doi: 10.1371/journal.pone.0242973 (PMC7707470; doi:10.1371/journal.pone.0242973)
Supplement: Attachment — Submitted filename: Response to Reviewers.docx [file pone.0242973.s007.docx]

**We would like to thank both reviewers for their very constructive and accurate feedback. We have now implemented their suggestions, which we feel have helped in greatly improving the paper.**

**Reviewer #1:**

**Short title: this should be a short form not a replica of the full title.**

*Proposed short title:*

*‘Characterisation of lower-limb muscles in older women’*

**Abstract
Line 29 “These variables were compared …” No need of this statement here. It looks like repetition of your aim “… their variability in a group of post-menopausal women”**

*This sentence has been removed and the aim has been extended as follow:*

*“This study hence aimed at characterising muscle volume, length and physiological cross-sectional area (PCSA) and their variability, between body sides and between individuals, in a group of post-menopausal women.”*

**Line 34 “Personalised maximal isometric force was then calculated …” is part of the methods.**

*This sentence has been moved to lines 30-31, where methods are described*

**Line 36 Your conclusion is not in line with your stated aim in the abstract.**

*Thank you, we agree with this comment and we rephrased the conclusion:*

*“In conclusion, this study showed for the first time anatomical asymmetry of lower-limb muscles in older women, suggesting that symmetry should not be assumed in this population. Furthermore, we showed that a scaling approach, widely used in musculoskeletal modelling, leads to an overestimation of the maximal isometric force for most muscles.*

**Line 42 “Key words” is one-word, Keywords. Arrange your keywords alphabetically and you may wish to limit the number to 5 to 7, which is enough.**

*We have modified the section accordingly:*

***“******Keywords: Biomechanics, Gait analysis, Muscle segmentation, Muscle volume, Musculoskeletal modelling, Musculotendon parameters, Physiological cross-sectional area (PCSA).”***

**Introduction
Line 84 “form” from**

*Thank you for noticing. This has now been edited as suggested.*

**Materials and Methods
Line 92 BMI should be written in full in the first instance. In the exclusion/inclusion criteria, do you consider the exercise history of the patients? If not, this should be stated as part of the limitation in the discussion.**

*We have now edited the sentence (lines 95-96):*

*“Exclusion criteria were: body mass index (BMI) <18 or >35, history of or current conditions…”*

*The exercise history of the participants was not contemplated as an exclusion criterion. Nonetheless, we do not believe that this represents a limitation: our findings did not highlight specific pattern in the measured muscle parameters which could have been associated with a possible exercise history. If the effects of a specific training were present in our data, these would have likely affected the measurements of entire functional muscle groups rather than individual muscles. Similarly, when comparing to literature data, we do not think that knowing the exercise history of our participants would have contributed to the understanding of our results. In fact, we had no information about the exercise history from the participants enrolled in the other in-vivo studies or from the cadavers in the dissection studies, therefore any consideration on this matter would have been pure speculation.*

*Incidentally we monitored weekly physically activity for 7/11 of our participants and they all were moderately active. Since this data was not available for all participants, we decided not to include it.*

**Line 99 “During …” This should be a new paragraph.**

*This was edited as suggested*

**Line 115 “Psoas muscle” Psoas major muscle? Or Psoas muscles were**

*We are indeed referring to Psoas major and we have now specified this in the text, line 120:*

*“The Psoas major muscle was removed…”*

**Line 116 “valuated” evaluated**

*Thank you, this has been corrected, line 121:*

*“Similarly, the foot extensors and flexors were not evaluated,”*

**Line 146 You need to state whether your data pass the normality test or not.**

*These sentences were added at lines 153-155:*

*“All variables were tested for normality using the one-sample Kolmogorov-Smirnov test in MATLAB and null hypothesis were then consistently tested using either a student’s t test in the case of normally distributed data or a Wilcoxon signed-rank test in the case of non-normally distributed data”*

*And lines 191-192:*

*“All investigated parameters were not normally distributed; therefore, non-parametric tests were selected for the various statistical analyses.”*

**Line 147 “MATLAB” version please.**

*This information has been included at the first occurrence line 141:*

*“All above computations were performed in MATLAB R2019b (The Mathworks Inc., Natick, MA, USA). V_M_ and l_M_ were then used to calculate the muscle PCSA according to (2): ”*

**Line 151 Did you employed any statistics (e. g one sample test) in comparing your results with that of literature? If not, this sentence is not relevant here.**

*We did not employ statistical tools to compare our values to literature. We agree with your comment, therefore we moved the sentence to lines 145-146.*

**Line 152 state the type of correlation e. g Pearson’s or Spearman rank order correlation etc.**

*The term correlation was improperly used here. It has been replaced with:*

*“Linear regressions were computed between…”*

*Figure 3 caption has been modified as well*

***Fig. 3 – Linear regression between muscle volume and anthropometric parameters.***

*Linear regression and coefficients of determination (R^2^) between total lower-limb muscle volume and body mass (left), height (middle-left), lower-limb mas (middle-right), BMI (right).*

**Line 153 use the abbreviated form of body mass index here.**

*Thank you, this has been modified, line 160:*

*“…and lower-limb mass, body mass, height, and BMI.”*

**Line 158 the significant level should be set at P < 0.05.**

*Our significance level α was set equal to 0.05 in the different statistical analyses. Null hypothesis was rejected when p value was below 0.05, as correctly stated by the reviewer. We have now clarified this as follow:*

*“Significance level α was set to 0.05 for all statistical tests.”*

**Results
Line 161 delete “As expected”**

*We have modified accordingly:*

*“The inter-operator analysis provided overall higher CoV….”*

**Lines 168 to 182, Table 1 under the body segment, thigh. This should be thigh and gluteal. Body segments and Muscles in plural forms***.*

*Thank you, we have edited the table as suggested*

**Discussion
Lack of control population for young adults may need to be stated as additional limitation of the study. Since it is clear that the subject’s characteristics varied between the present study and compared population in the literature.**

*Thank you for the comment, we agree this point should be touched in the discussion. We have now included the following paragraph at lines 357-360:*

***“****Our results suggest that muscle asymmetry could be higher in older adults due to age-related processes. However, this finding is based on comparison to literature [24], where data were obtained following a slightly different methodology. Therefore, a wider study, including a control group of younger women, should be designed to prove our hypothesis.* ***”***

**Despite the time-consuming nature of the muscle segmentation techniques, larger sample size may be needed to give better picture of the observed results. This may be added as recommendations.**

*Thank you, we have now discussed this aspect, see lines 356-357:*

*“The cohort enrolled for this study* *included eleven participants; a larger sample size would be needed to ensure generalisability of the results observed here.”*

**Conclusion
The first few sentences should be based on your results and in line with your objectives. You can conclude on the implications of your study. Avoid citation unless necessary.**

*Thank you, we agree with your comment and we have removed citations and rephrased accordingly (lines 367-369):*

***“****In conclusion, this study uniquely proved the existence of significantly large muscle- and subject-specific asymmetry in muscle volume, length, and PCSA. This suggests that individual differences in muscle geometry must not be neglected and inter-limb symmetry cannot be assumed in older women.****”***

**Line 355 “Acknowledgment” Acknowledgments**

*Edited as suggested.*

**-----------------------------------------------------------------**

**Reviewer #2:**

**Abstract:
- Line 23: “The ability of muscles to produce force depends on”, not by their anatomical features. Do not use shortages (it’s).**

*The sentence has been edited as suggested:*

*“The ability of muscles to produce force depends, among others, on their anatomical features and it is altered by ageing-associated weakening.”*

**- Line 29: “between left and right limbs”**

*This sentence was removed in response to the first reviewer’s comment*

**- Line 32-33: “Generally, muscle parameters were similar … but volumes were smaller”, otherwise we would expect the volumes to be similar, too.**

*Thank you, we have modified the sentence accordingly*

*“Generally, muscle parameters were similar to previous literature”*

**Introduction:
- Line 72: it is not clear what “the ratio of which” refers to, please clarify this sentence.**

*We have now clarified the sentence:
“The ratio of muscle volume and length is proportional to the muscle PCSA [25] and hence to the maximal isometric force a muscle can generate [26]..”*

**- Line 84: from instead of form.**

*Thank you for noticing this. It has now been corrected*

**Methods:
- The authors should explain why they chose women with osteopenia or osteoporosis (line 89-92) and mention it both in the abstract and the discussion. Do the authors expect any possible impacts on their results or not and if yes, what could they be like?**

*Thank you for this comment. The cohort was enrolled as part of a larger study focussing on osteoporosis and prevention of risk of fracture. However, in this study, we only looked at the muscle characteristics, without assuming connections to osteopenia or osteoporosis. We could not think of any possible impact that a change in bone mineral content or other features of osteoporosis could have on the muscle features here investigated. For this reason, we removed any reference to osteoporosis from the manuscript (lines 90-92).*

*“**Eleven post-menopausal women (mean (standard deviation, SD): 69 (7) y. o., 66.9 (7.7) kg, 159 (3) cm) with no movement limitations were recruited by the Metabolic Bone Centre, Northern General Hospital in Sheffield, UK as part of larger studies (Multisim and Multisim 2, EP/K03877X/1 and EP/S032940/1, https://epsrc.ukri.org).”*

**- Line 130-135: In our opinion the muscle length could have been calculated with a 3D extraction of the centreline instead of extrapolating it from filtered centrelines calculated on the 2D slices. Why was the muscle length not calculated directly from the 3D data? For example, with** [**https://www.mathworks.com/matlabcentral/fileexchange/43400-skeleton3d**](https://www.mathworks.com/matlabcentral/fileexchange/43400-skeleton3d) **or** [**https://www.mathworks.com/matlabcentral/fileexchange/71766**](https://www.mathworks.com/matlabcentral/fileexchange/71766)

*Our initial approach to the problem was indeed in line with what suggested here by the reviewers.*

*In fact, ‘Skeleton3D’ tool and ‘GPU Centreline Extraction’ tool were initially tested, but the results were sensitive to the shape of the specific muscle analysed. In detail, using the ‘Skeleton3D’ tool some muscle centrelines resulted in branched centrelines (regardless of the branch filter chosen), whereas, using the ‘GPU Centreline Extraction’ tool some centrelines resulted in disjointed lines. This affected, and in some cases impeded, the calculation of the length of the centreline. On the contrary, the selected 2D approach presented the advantage of being insensitive to the shape of the muscle and could therefore be applied to all the muscles consistently.*

**- The section about intra- and inter-operator repeatability should be clarified. Under which criteria was the intra-operator variability performed additionally and which approach was used for which muscle? This could for example be indicated in Table 1. Why was the cut-off for exclusion of a muscle set at 10%?**

*Thank you for this comment. We agree we did not clearly explain our methods and criteria.*

*Muscles for which an Inter-operator CoV of 5%, indicating good to excellent repeatability (Campbell et al., 2010) was found, were deemed as satisfactory to proceed with a multi-operator study (under the assumption that intra-operator results would be equal or better). The remaining muscles were further tested to quantify intra-operator repeatability and ensure that the analysis could provide reliable results at least when performed always by the same operator. Following the intra-operator analysis, we considered all the muscles having a CoV higher than 10% as highly unreliable and fully discarded them even when segmented by the same operator, (Wang et al. 2002).*

*We have now tried to clarify this	in the text:*

*“According to literature suggestions [30, 31], values of CoV can be considered as acceptable when below 10%. Using a conservative approach, for those muscles where inter-operator CoV was higher than 5% we also performed an intra-operator analysis, asking the same operator to repeat the segmentation three times on the same dataset. Following the latter analysis, we discarded all the muscles with non-acceptable repeatability (CoV > 10%).”*

*References:*

*Campbell, Michael J., David Machin, and Stephen J. Walters. Medical statistics: a textbook for the health sciences. John Wiley & Sons, 2010.*

*Wang CY, Olson SL, Protas EJ. Test-retest strength reliability: hand-held dynamometry in community-dwelling elderly fallers. Archives of physical medicine and rehabilitation. 2002 Jun 1;83(6):811-5.*

**- The ± sign should be specified in the text; does it mean standard deviation (SD) or range? Or in which case does it mean what? For example, line 88 about the age, weight and height of the subjects: as readers, we assume this to be the range, however, compared to the numbers in Fig. 3, it does not seem to be the case.**

*Thank you for this comment. We have now report standard deviations with brackets instead of the ± sign. This has been explained (lines 90-91):*

*“Eleven post-menopausal women (mean (standard deviation, SD): 69 (7) y. o., 66.9 (7.7) kg, 159 (3) cm) were recruited by the …”*

*Tables and captions have been modified accordingly (see Figures and tables section at the end of the document).*

**- Line 150: how was the CoV calculated?**

*As per its definition, CoV was calculated as the ratio between SD and mean. This is now defined in the manuscript:*

*“…was assessed by calculating the ratio between SD and mean (referred to as coefficient of variation, CoV) of the…”*

**- The abbreviation of “muscle volumes and length” is not the same throughout the text (line 122: VLS, line 130: MVLS). The authors should take care to always use the same abbreviation for the same term.**

*Thank you for spotting this mistake. We have now uniformed the abbreviations using VLS throughout the whole manuscript.*

*“In the VLS approach, muscle segmentations…”*

**- A citation or details for the MultiSim Study (line 89) should be given.**

*Thank you, we have now included a reference and link to the project at line 92*

*“…(Multisim and Multisim 2, EP/K03877X/1 and EP/S032940/1, https://epsrc.ukri.org)...”*

**- Line 91: the abbreviation DXA is not necessary, since the term appears only once in the whole manuscript.**

*Thank you for noticing. This has now been removed.*

**Results/Figures:
- Fig. 1, 2, and 4: switching column 2 and 3 (“Right” and “Left”) might increase the readability of the figures, since the inter-limb difference in % (column 4) is presented in this way.**

*We have implemented this suggestion, see Figures and tables section at the end of the document.*

**The authors should use the same notation for range in the whole figure and all the figures (either with a comma or a hyphen, if possible, also not use square brackets).**

*We have edited the table accordingly: we now use brackets and semicolon for the range.*

**In the text of the results the authors mention the between-subject CoV (line 190 for the muscle volume and line 192 for the muscle length), but they do not state which numbers belong to the volume and which to the length.**

*The CoV at line 190 (now line 197) is referring to volume and the CoV at line 192 (now line 200) is referring to length. We have now clarified this adding separated paragraphs for the results related to volume and those related to length:*

*“The percentage difference of V_M_ between the two limbs was above 85% in two subjects for the Gracilis and Rectus femoris, respectively. A significant difference between the two limbs was found for the V_M_ of the Sartorius, Gluteus maximus, Adductor magnus, and Vastus lateralis, with lower values in the left limb. Between-subject CoV (see supplementary material) ranged between 14% (Vastus medialis) and 36% (Sartorius).*

*The percentage difference of l_M_ between the two limbs was up to 22% (Adductor brevis). A significant difference between the two limbs was observed for the l_M_ of the Gluteus medius and Vastus lateralis, with lower values in the left limb. Between-subject CoV (see supplementary material) ranged between 3% (Sartorius) and 13% (Gastrocnemius lateralis).”*

**Furthermore, the numbers for the between-subject CoV from the text are not derivable from the numbers in the figures, the authors should clearly indicate which column shows the between-subject CoV in the figures.**

*Between subject CoV was not reported in the figures and was calculated as the ratio between the inter-subject SD and mean for each of the muscles. We have now reported this value as additional material and referenced it in the text (see previous answer).*

**- Figure 6: what does the p value in column 4 refer to? Does it show significance in the difference between LLMS and VLS? Then this should be mentioned in the figure legend and in the methods section, where the authors only state that the Wilcoxon test was used to show significance in limb asymmetry.**

*Thank you for the comment. We agree that this information was missing in caption and have now been included (lines 267-271):*

*“Median (minimum, maximum) of the maximal isometric force for the VLS and LLMS approaches with p values representing the statistical significance of Wilcoxon test.”*

*We have also clarified this point in the Methods section (lines 153-155):*

*“All variables were tested for normality using the one-sample Kolmogorov-Smirnov test in MATLAB and null hypothesis were then consistently tested using either a student’s t test in the case of normally distributed data or a Wilcoxon signed-rank test in the case of non-normally distributed data.”*

**Adjusting the presentation of the p values according to the other figures would be favourable (using * and ** instead of providing numbers).**

*The choice of using p values emerged from the fact that, differently from the previous figures, where statistical significance was reached only for few muscles, in this case most muscles presented significant difference.*

**Furthermore, the discussion of those results (line 315-316) should be clarified. If we understand it right, the muscles mentioned there are not the muscles with no significant difference, but the ones with higher values calculated with the VLS approach.**

*Indeed, the reviewer understood correctly. We have clarified the sentence (lines 329-331):*

*“Estimated F_max_ were overall significantly smaller when based on V_M_ than when linearly scaled to lower-limb mass, except for the Adductor magnus, Vastus intermedius and medialis, Gastrocnemius medialis, and Soleus, that, on the contrary, presented significantly higher values for the LLMS approach.”*

**- Table 2 (line 213) could be better understandable if the authors would indicate clearly which study was based on living subjects or cadavers and which used MRI or dissection.**

*We agree with your comment and we included information about the type of study in the table.*

**Why is the study by Ward et al. the only one without SD? Since the numbers seem to have been calculated by the authors (on a quick glance they do not seem to appear in the original work by Ward et al.), it would be nice if they could also provide the SD.**

*We have calculated muscle volumes from Ward et al.’s work by dividing the reported muscle mass by the muscle density as suggested in their study. We have now included this information. (lines 227-228)*

*“muscle mass values reported by Ward et al. [25] were multiplied by a muscle density of 1.056 g/cm^3,^ as suggested by the authors in [36].”*

*We have now included SDs for Ward’s muscle volume values as suggested.*

**Also, using Vm for muscle volumes generated specifically by MRI (as opposed to dissection) in this table is confusing since it usually stands for muscle volume.**

*We have replaced V_M_ with V_MRI._*

**- Line 187/188: the sentence sounds as if the percentage differences of Vm in the Gracilis and Rectus femoris were higher than 85% in all the subjects, however it is the case only in one subject for each of the muscles. The authors should rephrase this sentence.**

*Thank you, we agree that the sentence was misleading. It has now been rephrased (lines 194-195):*

*“…above 85% for the Gracilis in one subject and for the Rectus femoris in another subject.”*

**- Fig. 3 legend: Please complete the text according to the results, describing correlations and indicating significant values. Correct lower-limb mass (line 222).**

*The caption has been modified accordingly (lines 231-233):*

*“****Fig. 3 – Linear regression between muscle volume and anthropometric parameters.***

*Linear regression and coefficients of determination (R^2^) between total lower-limb muscle volume and body mass (R^2^ = 0.50, p = 0.003, left), height (R^2^ = 0.02, p > 0.05, middle-left), lower-limb mass (R^2^ = 0.14, p > 0.05, middle-right), BMI (R^2^ = 0.44, p = 0.004¸ right).”*

**- Fig. 5 legend: what does the figure show? Please complete the text. Correct PCSA values (line 241).**

*We have integrated the caption as it follows (lines 251-255):*

*”****Fig. 5 – PCSA distribution and comparison to literature data.***

*Distribution of the PCSA for the 22 limbs analysed in this study (grey violin plots) compared to PCSA values from literature. Red circles represent individual data points for three cadavers as calculated by Charles et al. [27]. Blue diamonds represent mean PCSA values for twenty-one cadavers as calculated by Ward et al. [25] and divided by the cosine of the mean pennation angle reported by the same authors. Green squares with deviation error bars represent PCSA values estimated by Handfield et al. [24] from MRI segmentation of thirty-two healthy young adults.”*

**- Fig. 5: Separating the data points from the three presented studies with a jitter or separation would increase the readability. Furthermore, it would be useful to indicate whether data points represent single subjects or mean values.**

*As suggested, we have indicated in the caption whether data points represent individual values or means (see answer to previous comment).*

*We have now used a randomised value (similarly to a jitter-based approach) to separate the data points and improve readability of the figure. Please note that the current figure is only meant to support a visual comparison between the data obtained from our study and literature data. Specific values of PCSA calculated in our study are available in Fig.4*

**- Line 224: “(fig. 4)” needs to be formatted consistently with other figure mentions.**

*Thanks for noticing, we have corrected the error:*

*“The percentage difference in PCSA (Fig. 4) between…”*

**Discussion:
- In order to follow the flow of the discussion it might be useful to adhere to the order of the results and figures and to reference the figure which shows the respective results.**

*Thank you for this comment. We have now included cross-references to figures and tables in the manuscript.*

*We agree that following the same order as in the Results section facilitates the reader. For this reason, the paragraph about regression equations has now been moved to lines 304-312 before the paragraph about maximal isometric force.*

**- Since the calculation of Vtot consisted of only 23 muscles, the authors should discuss this as an additional possible reason why it did not correlate with the lower-limb-mass.**

*We agree that considering only 23 muscles could limit the correlation of Vtot to lower-limb mass, but similarly should affect the correlation to body mass. In our discussion we comment on the higher correlation of Vtot to body mass compared to lower-limb mass. Despite having considered only 23 muscles we would have expected to see a stronger correlation for the lower-limb mass.*

*“Surprisingly, V_TOT_ correlated more strongly with total body mass than with lower-limb mass… ”*

*For this reason, we do not believe that the Discussion would benefit from additional comments on the matter.*

*When, instead, discussing the correlation to anthropometric parameters and comparing to previous literature, we indeed acknowledge that the smaller number of muscles included in our study could be a limitation. In fact we commented on this in the text:*

***“****Lower correlations were quantified in this study, likely due to having included only 23 instead of 35 lower-limb muscles.****”***

**- It would be interesting to know if there is a possible explanation for the asymmetric left/right distribution of the muscle volume and length. Is there any recognisable pattern, e.g. that some individuals are in fact rather left- than right-footed, or could it be the case that smaller muscles in one functional group are being compensated by larger volumes of the other muscles in the same group?**

*Thank you for the interesting comment, we did investigate limb dominance in order to look for recognisable patterns or correlations to volume asymmetry, however we could not identify any consistent pattern.*

*We have now reinforced this in the discussion (lines 287-288):*

*“no recognisable* *pattern was observed across the cohort that could be associated* *with limb dominance.”*

*We also looked at possible compensation mechanisms between muscles in the same functional group by summing the volumes of the muscles in the same functional group, but also in this case we observed asymmetry ranging between 22% (larger right knee extensors) and -25% (larger left knee flexors). We could not identify any pattern for the asymmetry of the various muscle groups in the different participants. In fact, despite all the subjects were right dominant, only one subject showed consistent, but not significant, larger volumes for all the muscle groups of the right limb.*

*Perhaps a larger cohort could provide more information, as also discussed as a limitation of the study.*

**Could additional anamnestic info about hip/knee problems or movement limitations be helpful to understand possible patterns?**

*In the current study we did not observe any movement limitations in the participants, suggesting that muscle asymmetry can happen in healthy older subjects with no recorded movement alteration.*

*We have now specified this in the manuscript (lines 90-91):*

*“Eleven post-menopausal women (mean (standard deviation, SD): 69 (7) y. o., 66.9 (7.7) kg, 159 (3) cm) with no movement limitations were recruited by the…”*

**- Line 328: “which proved to cause variation up to 400% in our study …"**

*This was edited:*

*“…account for actual muscle geometry to estimate F_max_, which proved to cause variation up to 400% in our study when compared to scaling approaches.”*

**- Grammar corrections: line 264 “However, …"; line 301 “Valente et al., finding ...”; line 306 “In order to overcome …”**

*We modified according to what has been noted (lines 278, 324, 304 ):*

*“However, broadening…”*

*“…previously tested by Valente et al. [13], finding…”*

*“In order to overcome the need for individual muscle segmentations…”*

**Acknowledgement:
- Line 356: from instead of form; line 358: grant numbers (since they are two); line 360: we assume that the “the views expressed” are the ones of all the authors, hence author(s) should be corrected.**

*The section has been modified accordingly:*

*“The authors would like to acknowledge Dr Geoffrey Handsfield for sharing relevant data from [24] and Dr Enrico Dall’Ara for his valuable input around image processing.*

*The study was funded by the UK EPSRC through the MultiSim and MultiSim2 projects (grant numbers EP/K03877X/1 and EP/S032940/1) and carried out at the NIHR Sheffield Biomedical Research Centre (BRC). The views expressed are those of the authors and not necessarily those of the NHS, the NIHR or the Department of Health and Social Care (DHSC). We are particularly grateful to the participants who volunteered for the study. ”*

***Figures and tables:***

**Table 1 – Repeatability of muscle segmentation**

| **Body segments** | **Muscles** | **Inter-op CoV** | **Intra-op CoV** |  |  |  |  |
| --- | --- | --- | --- | --- | --- | --- | --- |
| Thigh and gluteal | Iliacus | 8.0 | 2.6 |  |  |  |  |
|  | Sartorius | 10.2 | 2.0 |  |  |  | CoV ≥ 10% |
|  | Gluteus maximus | 7.0 | 2.0 |  |  |  | CoV < 10% |
|  | Gluteus medius | 10.6 | 5.3 |  |  |  | CoV < 5% |
|  | Gluteus minimus | 14.6 | 21.6 |  |  | - | Not tested |
|  | Tensor fasciae latae | 12.4 | 1.1 |  |  |  |  |
|  | Adductor brevis | 22.8 | 7.5 |  |  |  |  |
|  | Adductor longus | 17.7 | 6.0 |  |  |  |  |
|  | Adductor magnus | 5.9 | 3.6 |  |  |  |  |
|  | Gracilis | 16.1 | 2.7 |  |  |  |  |
|  | Biceps femoris long head | 7.6 | 4.7 |  |  |  |  |
|  | Biceps femoris short head | 9.9 | 4.7 |  |  |  |  |
|  | Semimembranosus | 9.7 | 6.9 |  |  |  |  |
|  | Semitendinosus | 6.9 | 5.2 |  |  |  |  |
|  | Rectus femoris | 7.0 | 5.6 |  |  |  |  |
|  | Vastus intermedius | 6.6 | 1.1 |  |  |  |  |
|  | Vastus lateralis | 9.8 | 1.2 |  |  |  |  |
|  | Vastus medialis | 4.2 | - |  |  |  |  |
| Calf | Tibialis anterior | 25.3 | 4.2 |  |  |  |  |
|  | Tibialis posterior | 12.1 | 8.9 |  |  |  |  |
|  | Gastrocnemius lateralis | 4.6 | - |  |  |  |  |
|  | Gastrocnemius medialis | 4.5 | - |  |  |  |  |
|  | Soleus | 8.6 | 5.9 |  |  |  |  |
|  | Peroneus brevis | 49.4 | 7.6 |  |  |  |  |
|  | Peroneus longus | 48.2 | 10.9 |  |  |  |  |

Inter- and intra-operator coefficient of variation (CoV) for muscle volume calculated by three operators (inter-op) and by one operator over three repetitions (intra-op).

**Fig. 1 – Muscle volume variability.**

Median (minimum, maximum) of muscle volume for the right and left limb (significant difference between limbs: * p<0.05, **p<0.01). Individual percentage difference between the limbs is reported as a bar plot where each bar represents a participant: blue positive (red negative) values show that the right leg is bigger (smaller). Minimum and maximum percentage difference across the subjects is reported for each muscle.

**Fig. 2 – Muscle length variability.**

Median (minimum, maximum) of muscle length for the right and left limb (significant difference between limbs: **p<0.01). Individual percentage difference between the limbs is reported as a bar plot where each bar represents a participant: blue positive (red negative) values show that the right leg is bigger (smaller). Minimum and maximum percentage difference across the subjects is reported for each muscle.

**Table 2. Comparison of muscle volume to literature values.**

Mean (standard deviation, SD) of the muscle volume for a subset of lower-limb muscles for the eleven subjects in the current study. V_MRI_ (grey columns) are the volumes obtained from MRI segmentation calculated in our study, in Handsfield et al. [24] from eight healthy young females (30 (8) y.o.), and in Charles et al. [27] from three cadavers (36 (14) y.o.). V_D_ are the muscle volumes obtained from cadaver dissection from three cadavers by Charles et al. [27] and from twenty-one elderly cadavers (83 (9) y.o.) by Ward et al. [25]. * with participants we here refer to the number of limbs considered independently; gender of the participants is reported too. † muscle mass values reported by Ward et al. [25] were multiplied by a muscle density of 1.056 g/cm^3^ as suggested by the authors in [36].

**Fig. 3 – Linear regression between muscle volume and anthropometric parameters.**

Linear regression and coefficients of determination (R^2^) between total lower-limb muscle volume and body mass (R^2^ = 0.50, p = 0.003, left), height (R^2^ = 0.02, p > 0.05, middle-left), lower-limb mass (R^2^ = 0.14, p > 0.05, middle-right), BMI (R^2^ = 0.44, p = 0.004¸ right).

**Fig. 4 - PCSA variability.**

Median (minimum, maximum) of the physiological cross-sectional area (PCSA) for 23 lower-limb muscles for eleven subjects in our study (n = number of limbs). PCSA are derived from the segmented V_M_ and l_M_ and using the average optimal fibre length to muscle length ratio proposed by Ward et al. [25]; *PCSA of the Tensor fasciae latae was calculated setting the optimal fibre length to muscle length ratio equal to 1 (as proposed by Handsfield et al. [24]) since the actual values were not available from the literature source. Minimum and maximum percentage difference across the subjects is reported for each muscle.

**Fig. 5 – PCSA distribution and comparison to literature data.**

Distribution of the PCSA for the 22 limbs analysed in this study (grey violin plots) compared to PCSA values from literature. Red circles represent individual data points for three cadavers as calculated by Charles et al. [27]. Blue diamonds represent mean PCSA values for twenty-one cadavers as calculated by Ward et al. [25] and divided by the cosine of the mean pennation angle reported by the same authors. Green squares with deviation error bars represent PCSA values estimated by Handfield et al. [24] from MRI segmentation of thirty-two healthy young adults.

**Fig. 6 – Maximal isometric force calculated with the VLS and LLMS approach.**

Median (minimum, maximum) of the maximal isometric force for the VLS and LLMS approaches with p values representing the statistical significance of Wilcoxon test. Individual percentage difference of F_max_ between VLS and LLMS reported as a bar plot where each bar represents a participant: green positive (orange negative) bars show that the value is bigger (smaller) with the VLS approach. Minimum and maximum percentage difference across the subjects is reported for each muscle.* These values correspond to one third of the total muscle F_max_
