# Supplementary material for: MRI-based anatomical characterisation of lower-limb muscles in older women
Source: PLoS One. 2020 Dec 1;15(12):e0242973. doi: 10.1371/journal.pone.0242973 (PMC7707470; doi:10.1371/journal.pone.0242973)
Supplement: Attachment — Submitted filename: Response to Reviewers.docx [file pone.0242973.s008.docx]

**We would like to thank the reviewer for this further feedback to our manuscript. We have now implemented the suggestions.**

**Reviewer #1:**

**Line 28 Lower limb or Lower-limb. Please be consistent**

*Thank you, have modified the manuscript "Lower-limb"*

**Line 102 “Declaration of Helsinki” Please give year**

*We have included the above information:*

*"The study was approved by the East of England - Cambridgeshire and Hertfordshire Research Ethics Committee and the Health Research Authority and was conducted in accordance with the Declaration of Helsinki (October 2000).* "

**Line 114 “SD” write in full.**

*Thank you for the comment, however standard deviation was already defined at a previous occurrence (line 90).*

Line “References” need formatting e.g. Journal names are mixed of Title case and Sentence case

*Thank you for noticing this. We have double checked the references and made sure we used Sentence case for titles and Title case for journal name.*
